# Feasibility of a Humor Training to Promote Humor and Decrease Stress in a Subclinical Sample: A Single-Arm Pilot Study

**DOI:** 10.3389/fpsyg.2018.00577

**Published:** 2018-04-24

**Authors:** Nektaria Tagalidou, Viola Loderer, Eva Distlberger, Anton-Rupert Laireiter

**Affiliations:** ^1^Department of Psychology, University of Salzburg, Salzburg, Austria; ^2^Faculty of Psychology, University of Vienna, Vienna, Austria

**Keywords:** humor training, subclinical, coping humor, cheerfulness, perceived stress, single-arm

## Abstract

The present study investigates the feasibility of a humor training for a subclinical sample suffering from increased stress, depressiveness, or anxiety. Based on diagnostic interviews, 35 people were invited to participate in a 7-week humor training. Evaluation measures were filled in prior training, after training, and at a 1-month follow-up including humor related outcomes (coping humor and cheerfulness) and mental health-related outcomes (perceived stress, depressiveness, anxiety, and well-being). Outcomes were analyzed using repeated-measures ANOVAs. Within-group comparisons of intention-to-treat analysis showed main effects of time with large effect sizes on all outcomes. *Post hoc* tests showed medium to large effect sizes on all outcomes from pre to post and results remained stable until follow-up. Satisfaction with the training was high, attrition rate low (17.1%), and participants would highly recommend the training. Summarizing the results, the pilot study showed promising effects for people suffering from subclinical symptoms. All outcomes were positively influenced and showed stability over time. Humor trainings could be integrated more into mental health care as an innovative program to reduce stress whilst promoting also positive emotions. However, as this study was a single-arm pilot study, further research (including also randomized controlled trials) is still needed to evaluate the effects more profoundly.

## Introduction

Increased levels of stress are highly prevalent (Wiegner et al., 2015) and entail serious physical and mental health problems. Prolonged stress increases the risk of acute myocardial infarction (Rosengren et al., 2004), weakens the immune system (Segerstrom and Miller, 2004), and is related to depression, exhaustion (Wiegner et al., 2015), and reduced quality of life (Golden-Kreutz et al., 2005).

Adaptive appraisal and coping can be good ways to handle stress more effectively and so diminish the negative impact of it on health outcomes (Lazarus and Folkman, 1984). However, not everybody possesses the ability to cope with stress adequately and suffers from its consequences. Due to that, stress prevention and stress reduction programs receive growing attention, as they teach the use of adaptive coping mechanisms and therefore help handle prolonged stress (Jaremko and Meichenbaum, 2013).

A new and promising strategy to handle and reduce stress, whilst furthermore promoting also mental health and well-being, is the use of humor. Humor has already been recognized as an effective stress moderator (Martin, 2011): Research shows that the use of humor is an adaptive emotion regulation strategy in the short-term (Strick et al., 2009; Samson and Gross, 2012; Kugler and Kuhbandner, 2015) and also serves as a coping strategy against negative and stressful life situations in the longer-term (Martin and Lefcourt, 1983; Labott and Martin, 1987; Overholser, 1992; Sliter et al., 2014). Furthermore, using humor does not only downregulate negative emotions but elicits also positive emotions, such as amusement (Herring et al., 2011), which are important for promoting resilience and well-being as stated by the broaden-and-built theory (Fredrickson and Joiner, 2002; Fredrickson, 2004).

Due to its basic working mechanisms (downregulation of negative emotions and upregulation of positive emotions) humor is positively related to life satisfaction, positive affect, and well-being (Martin et al., 2003; Martinez-Marti and Ruch, 2014), and has positive effects in various aspects of life (Martin, 2011).

To profit from the diverse positive effects of humor, latest research has focused on improving humor through various interventions, especially humor trainings. Humor trainings can differ in their structure and grounded theory; however, they all aim at the same targets: to promote positive emotions, longer-lasting positive mood states such as cheerfulness (Ruch et al., 1996), and most importantly coping humor, which is defined as the ability to use humor to cope with stress (Ruch and Hofmann, 2017). A widely known humor training program was developed by McGhee (1996, 2010) called the “7 Humor Habits program.” It has already proven its efficacy in increasing/decreasing several mental health outcomes like positive affect, life satisfaction, depression, and anxiety (Sassenrath, 2001, unpublished; Beh-Pajooh et al., 2010; Crawford and Caltabiano, 2011; Ruch et al., 2018). Also, most importantly, perceived stress and stress levels can indeed be reduced by the participation at the training (Crawford and Caltabiano, 2011).

It is important to note that the studies just mentioned used only healthy participants in their designs. Research on humor trainings in clinical settings is scarce, although people suffering from mental disorders would profit from them. Mental disorders can entail various humor- and stress-related deficits. Regarding humor, difficulties in cognitive and affective components of humor or difficulties experiencing cheerfulness have been reported (Uekermann et al., 2007; Uekermann et al., 2008; Falkenberg et al., 2011). Regarding stress, people with mental disorders show serious deficits in emotion regulation and coping processes (Aldao et al., 2010). The training could help affected people improve their sense of humor, so that they are able to use it for coping with stress and negative affectivity in daily life. Some studies already tried to investigate the effects of humor trainings in clinical populations and found promising results. Falkenberg et al. (2010), for example, could demonstrate an increase in coping humor for depressed inpatients and Cai et al. (2014) found improvements in symptomatology, depression, and sense of humor for schizophrenics. Tagalidou et al. (in press) found improvements in coping humor and cheerfulness for people of a routine care institution suffering from schizophrenia, personality disorders, anxiety, or depression. All studies used McGhee’s humor training. Further studies, using an alternative humor training program for depressed elderly inpatients, could also show positive results like changes in resilience, cheerfulness, or satisfaction with life (Hirsch et al., 2010; Konradt et al., 2013).

As can be seen, research on manualized humor trainings is relatively new. There have been studies conducted with healthy or clinical samples which show promising results. However, studies including participants with stress-related and subclinical, yet burdening, symptoms like increased levels of stress, depressiveness, or anxiety have not yet been published at all, although it would be reasonable to concentrate on this population, too. Subclinical problems can easily grow up to clinical symptoms which have to be treated with psychotherapy or psychotropic drugs, burdening affected people and the health care system (Vigo et al., 2016). A low-threshold, preventive offer like a short humor training could help decrease subclinical symptoms and stress and furthermore promote cheerfulness. By integrating both these aspects at an early stage of symptom development, ideally it would be possible to diminish incidence rates of mental disorders.

### Aims and Research Questions

The study investigates feasibility of a humor training for people with subclinical symptoms like increased levels of stress, depressiveness, or exhaustion and tries to narrow the gap opened by lack of research in this area. The main focus is the evaluation of the training as a low-threshold, preventive program against everyday life stress and hassles.

Although different stress preventive programs have already been developed to reduce stress by now, most of them concentrate mainly on the reduction of stress-related symptoms. The promotion of positive aspects like well-being, resilience, and personal strengths remains rather neglected. If the training appears to be as feasible as already broadly implemented programs [see e.g., “Mindfulness-Based Stress Reduction” (MBSR); Grossman et al., 2004; Khoury et al., 2015], it is conceivable to integrate humor trainings in future health-care systems as additional and alternative prevention programs against stress and mental disorders. Interventions like this one could help decrease the incidence rates of mental symptoms as they intervene already at early stages of symptom development and thereby also promote cheerfulness and well-being.

The study focuses on three main objectives: the first one is to evaluate if the humor training can improve humor related outcomes. The training mainly promotes using humor under stress (coping humor), therefore coping humor was chosen as a humor-related outcome. Furthermore, cheerfulness was assessed as a longer-lasting positive mood state.

The second aim is to evaluate if the training can improve mental health and well-being. To test this hypothesis, several mental health-related outcomes were included in the study’s design. As the training primarily tries to decrease stress, perceived stress was included as an outcome variable. Further, depressiveness and anxiety were included to test if the training can improve subclinical forms of depressiveness and anxiety. Lastly, well-being was included as a positive outcome of the training.

The third aim of the study is to evaluate applicability of the training based on the feedback of participants. Evaluation of feedback in humor training studies is scarce, so we want to emphasize this aspect more to get a broader overview about the feasibility of the training.

## Materials and Methods

### Design

The design of the study was a single-arm trial to explore feasibility of the humor training for a subclinical population currently experiencing increased stress, depressiveness, or exhaustion. The within-factors design had three measurement time points: 1 week before treatment, 1 week after treatment, and a 1-month follow-up after treatment.

The training took place in the outpatient clinic of the University of Salzburg. The study’s protocol was approved by the ethics commission of the University of Salzburg (44/2016) and registered in the German Clinical Trials Register (DRKS00013480).

### Participants

Calculated by G^∗^Power 3.1 (Faul et al., 2007), the required sample size to find a medium effect of *f* = 0.25 with a power of β = 0.80 and an α level of 0.05 was 28 (a medium effect was assumed for calculation based on reviewing already existent research on humor trainings). However, to also cover potential dropouts, a higher *N* than 28 is needed. McDermut et al. (2001) reported an average attrition rate of 18.6% for group therapies of mood disorders. Considering their result, a total *N* of 33 was assumed to be needed. In the end, the final number of participants was 35. They were recruited via an advertising article in the local newspaper of Salzburg (“Salzburger Nachrichten”), which contained a report about the planned humor training. The article addressed people who currently experienced stressful situations, depressiveness, or exhaustion in their daily lives and it was explained how humor and the experience of cheerfulness can help in coping better with negative situations and emotions. Everyone who was interested in humor and experienced stress and hassles in daily life was invited to participate in the study. Participants received the humor training for free; in return they were asked to fill in evaluation questionnaires. It was not explained which outcomes are being measured and analyzed. Participants were only told that the humor training helps coping better with daily life stress.

The inclusion criterion for participating in the study was the subclinical experience of symptoms like increased stress, exhaustion, depressiveness, or anxiety. Thus, if someone showed clinical symptoms and fulfilled the criteria for any current mental disorder, he or she was excluded from the study. Only one exception was made for people with a recurrent depressive disorder currently in remission (ICD 10: F33.4, DSM-IV: 296.36). These people do not show any symptoms of a current depressive episode; however, they generally experience subclinical depressive symptoms frequently and have high risk of recurrences (Solomon et al., 2000). So, to help them build up preventive strategies against forthcoming relapses, they were invited to participate in the study. Further inclusion criteria for the study have been good German language skills and no cognitive deficits like dementia.

**FIGURE 1:**
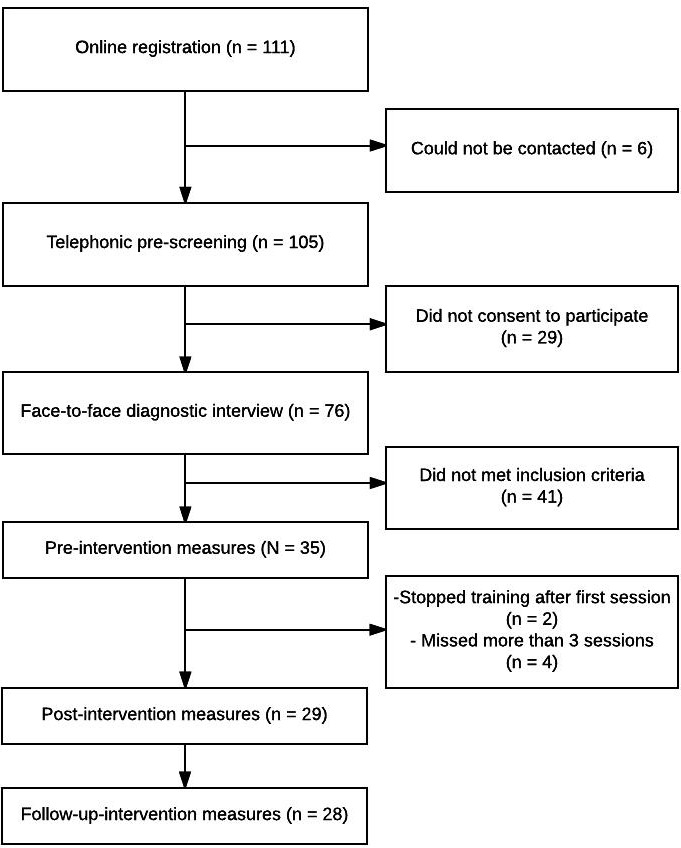
Flowchart of the study.

A total of 111 people were interested in the training and submitted registration. Of these, 105 (94.6%) could be contacted for the telephonic pre-screening. Seventy-six (68.5%) participated in the face-to-face diagnostic interview and finally 35 (31.5%) persons met inclusion criteria and started training. **Figure 1** depicts the complete selection process.

### Procedure

If interested, people registered online on the training’s homepage and submitted contact details so they could be contacted telephonically. During the phone call, general organizational information was communicated and a quick pre-screening, concerning interest and motivation for participation, was conducted. If the interested persons appeared to suit in the study’s design, they were invited to take part in a face-to-face diagnostic interview. The interview was conducted using the Structured Clinical Interview for DSM-IV, I, and II (Wittchen et al., 1997). Only people who showed no current mental disorder or a recurrent depressive disorder currently in remission were allowed to participate in the training. The interviews were conducted by employees of the outpatient clinic who have been in training as clinical psychologists. They got a regular training and had sufficient experience with diagnostic interviews in general, and the SCID I and II manual in specific.

Finally, people who fulfilled inclusion criteria were invited to participate in the training. They were assigned to a training group and written informed consent and a non-disclosure agreement were signed.

There were four humor training groups which started consecutively. Each group was led by two group leaders. In total, six group leaders conducted trainings. They were employees of the outpatient clinic and in training as clinical psychologists or trained master’s students at the end of their studies. They all had clinical experience and were extensively introduced to and trained in the humor training program. Assignment of trainers to the groups was random.

### Humor Training

The humor training is based on the German manual of Falkenberg et al. (2013). It is a 7-week program to promote cheerfulness and humor in everyday life and based on McGhee’s (1996, 2010) “7 Humor Habits Program”. Special attention is paid to the improvement of coping humor abilities, so that participants can use humor as a protective factor against personal stressful situations. The manual of Falkenberg et al. (2013) was developed specifically for people with mental disorders. However, we still used it for our subclinical population as the contents can easily be transferred also to people not suffering from a mental disorder. Furthermore, we slightly modified the manual with our own ideas so that it was more suitable for our sample. The training contained psychoeducational elements, which were combined with various exercises like role plays, games, and discussions. Every session addressed one specific humor topic like finding humor in everyday life, promoting playfulness, and finding a benevolent attitude toward personal weaknesses. Every session lasted 90 min. Additionally, participants had to do homework to implement the learned better in everyday life. **Table 1** summarizes the seven sessions and their associated content.

**Table 1:** Topics of the humor training.

Session	Content
1. Session	• Topic: Introduction
	• Meet and greet
	• Definitions of humor
	• The own sense of humor
2. Session	Topic: Seriousness vs. playfulness
	• The effects of seriousness on everyday life
	• The function of play and playful behavior
3. Session	Topic: Laughter
	• Physical and mental benefits of laughter
	• Duchenne vs. non-Duchenne laughter
	• Laughter exercises
4. Session	Topic: Creating verbal humor
	• Telling jokes
	• Ambiguousness
	• Exaggeration
5. Session	Topic: Finding humor in everyday life
	• Change of perspectives
	• Searching for humor consciously in daily situations
6. Session	Topic: To laugh about oneself
	• Humorous perspective on personal weaknesses
	• Pros/cons of laughing at oneself
7. Session	Topic: Finding humor in stressful situations
	• Definition of stress
	• Effects of humor on stress
	• Feedback

To get a better overview of the sessions’ structure, session 3, “laughter” will be explained in more detail. The session starts with an opening game to activate the participants and get them into positive mood. After that, homework is discussed and the last session briefly summarized. Beginning with the psychoeducational part of the session, the positive effects of laughter on physical and mental health and the concept of real (Duchenne) vs. fake (non-Duchenne) smiles are explained. People then participate in a quiz, where they have to detect Duchenne or non-Duchenne smiles on their own. After the general information about laughter and smiling, participants have an imagination exercise about laughter and group work, where they have to make their partner laugh with funny grimaces or jokes. In the end, homework is discussed and a funny closing game played.

### Measures

All outcomes were measured online using self-report questionnaires. Additionally, a feedback questionnaire was included after training, which could be filled in voluntarily and anonymously by participants.

#### Humor-Related Outcomes

Coping humor was measured using the *Coping Humor Scale (CHS)* by Martin and Lefcourt (1983). It is an economical 7-item scale which assesses the amount of humor someone uses to cope with stressors. The 4-point Likert scale ranges from 1 to 4. Internal consistency (Cronbach’s alpha) was between α = 0.75 and 0.80 for the three measurement time points.

Cheerfulness was measured using the *State-Trait-Cheerfulness Inventory (STCI)* – state version, which assesses short-term changes of exhilaration (Ruch et al., 1996, 1997). The questionnaire has three subscales: cheerfulness, seriousness, and bad mood with 10 items each and a 4-point Likert scale (1–4). Internal consistencies were between α = 0.90 and 0.93, α = 0.62 and 0.81, and α = 0.88 and 0.93 for the three scales and measurement time points respectively.

As an additional humor-related outcome, which will be analyzed only descriptively, gelotophobia was assessed before treatment using the *Gelotophobia Questionnaire (GELOPH-15*) by Ruch and Proyer (2008a,b). Gelotophobia is defined as the fear of being laughed at by others (Ruch and Proyer, 2008a,b) and the questionnaire was included to get a more detailed picture about the characteristics of the sample. As the training contains numerous situations with laughter and cheerfulness and would stress people with gelotophobia, it is interesting to explore how many people with gelotophobic fears would in fact register for humor training. Ruch and Proyer (2008a,b) have defined three cut-off criteria for gelotophobia: A mean ≥2.50 indicates a slight degree, a mean ≥3.00 a marked degree, and a mean ≥3.50 an extreme degree of gelotophobia. Fifteen items with a 4-point Likert scale (1–4) show an internal consistency of α = 0.87 at pre-treatment.

#### Mental Health-Related Outcomes

Perceived stress was assessed with the German version of the well-established *Perceived Stress Scale (PSS)* by Klein et al. (2016). Ten items with a 5-point Likert scale (0–4) show an internal consistency between α = 0.76 and 0.81 for the three measurement time points.

To evaluate the changes in depressive symptoms, the German *Center for Epidemiological Studies Depression Scale Revised (CESD-R)* was used. It was developed by Hautzinger et al. (2012) and includes 15 items with a 4-point Likert scale (0–3). Internal consistency was between α = 0.74 and 0.87 for the three measurement time points.

Anxiety was measured using the German translation of the *State-Trait-Anxiety Inventory (STAI)* in the state version (Laux et al., 1981). It consists of 20 items with a 4-point Likert scale (1–4) and its internal consistency was between α = 0.89 and 0.94 for the three measurement time points.

The German version of the economic *WHO-5 Well-Being Index (WHO-5)* was used as a screening tool for subjectively perceived well-being (Brähler et al., 2007). The five items have a 6-point Likert scale (0–5) and an internal consistency between α = 0.73 and 0.79 for the three measurement time points.

#### Evaluation of Applicability

A feedback questionnaire with 14 quantitative items and three qualitative items was constructed by the authors to evaluate the general satisfaction and applicability of the training. The quantitative items range from 1 to 5 except the last question “Would you recommend the training?” which ranges from 1 to 4. All items were analyzed separately on the item level.

### Statistical Analyses

The statistical software used was JASP 0.8.4 (JASP, 2017) and all analyses were calculated based on the intention-to-treat technique (ITT). Missing data at post and follow-up were imputed with the last observation carried forward method (LOCF). Outcomes were analyzed using repeated-measures ANOVAs. Time main effects and *post hoc* tests with Bonferroni correction (pre–post and pre–follow-up) were calculated. Furthermore, effect sizes of time main effects and *post hoc* tests were analyzed to evaluate the effects of the training more profoundly. Cohen’s *d* was chosen as effect size for the time main effects which was converted from eta squared (η^2^) based on the formula of Cohen (1988). Effect sizes of *post hoc* tests with 95% confidence interval are also reported in Cohen’s *d* based on the formula of Gibbons et al. (1993). Cohen (1988) defines *d* = 0.2 as small effect, *d* = 0.5 as medium effect, and *d* = 0.8 as large effect. Feedback was analyzed descriptively for quantitative items and qualitatively for open-format items.

## Results

### Sample Characteristics

**Table 2** summarizes demographic characteristics of the sample. Generally, participants had a mean age of 51.9 years (*SD* = 9.67), were predominantly female (*n* = 26, 74.3%), and Austrian (*n* = 33, 94.3%). They were mainly well educated (*n* = 26, 74.3%) and employed (*n* = 22, 62.9%).

**Table 2:** Demographic characteristics of the sample (*N* = 35).

	*M* (*SD*) or *n* (%)
**Age, *M* (*SD*)**	51.9 (9.67)
**Gender, *n* (%)**	
Female	26 (74.3%)
Male	9 (25.7%)
**Nationality, *n* (%)**	
Austrian	33 (94.3%)
German, Romanian, Swiss, Lithuanian (1 in each case)	4 (11.6%)
**Education, *n* (%)**	
≥9 years of education (compulsory school)	9 (25.7%)
≥12 years of education (A level)	11 (31.4%)
≥ any tertiary education (e.g., university)	15 (42.9%)
**Employment**	
Currently employed	22 (62.9%)
Retirement	6 (17.1%)
Parental leave/educational leave	3 (8.6%)
Partial retirement	2 (5.8%)
Student	1 (2.9%)
Not specified	1 (2.9%)
**Subclinical symptoms**	
Without *F* 33.4/ 296.36	28 (80.0%)
With *F* 33.47 296.36	7 (20.0%)
**Treatment**	
Psychotherapy	1 (2.9%)
Psychotropic drugs	6 (17.1%)
**Gelotophobia**	
No degree (<2.50)	33 (94.3%)
Slight degree (≥2.50)	1 (2.9%)
Marked degree (≥3.00)	1 (2.9%)

With regard to inclusion criteria, 28 persons (80.0%) reported subclinical symptoms without depressive episodes in the past and 7 people (20.0%) reported subclinical symptoms with a recurrent depressive disorder currently in remission. One person was in psychotherapy (2.9%) and six took psychotropic drugs (*n* = 6, 17.1%). While participating in the study, two people (5.7%) had changes in their medication. One person changed dose and one person discontinued medication. Gelotophobic fear has been small in the sample: only one person reported a slight and another person a marked degree of gelotophobia. Thirty-three people were below the cut-off score of 2.50.

At the end, dropout of the study was similar compared to the average attrition rate of 18.6% (McDermut et al., 2001). Two persons (5.7%) stopped training after the first session and 4 persons (11.4%) missed three or more sessions and were therefore classified as non-completers (total attrition rate: 17.1%).

**Table 3** summarizes mean values, standard deviations, and effect sizes for the outcomes at pre, post, and follow-up.

**Table 3:** *M*, *SD*, and effect sizes (pre–post and pre–follow-up) for the ITT analysis of outcome measures (*N* = 35).

	Pre *M* (*SD*)	Post *M* (*SD*)	Follow-up *M* (*SD*)	Pre–post effect size (Cohen’s *d*)	Pre–follow-up effect size (Cohen’s *d*)
Coping humor (CHS)	2.47 (0.58)	2.80 (0.52)	2.77 (0.57)	0.88 [0.48–1.27]^∗∗∗^	0.63 [0.26–0.99]^∗∗^
Cheerfulness (STCI)	2.13 (0.54)	2.62 (0.61)	2.72 (0.62)	0.75 [0.37–1.12]^∗∗∗^	0.88 [0.49–1.28]^∗∗∗^
Seriousness (STCI)	2.99 (0.36)	2.64 (0.44)	2.54 (0.52)	0.80 [0.42–1.18] ^∗∗∗^	0.91 [0.51–1.30]^∗∗∗^
Bad mood (STCI)	2.07 (0.66)	1.66 (0.53)	1.59 (0.64)	0.61 [0.24–0.96]^∗∗^	0.65 [0.28–1.01]^∗∗∗^
Perceived stress (PSS)^a^	19.46 (5.47)	14.80 (4.63)	14.26 (4.06)	1.05 [0.63–1.46]^∗∗∗^	1.09 [0.66–1.50]^∗∗∗^
Depressiveness (CES-D)^a^	12.63 (5.49)	8.03 (4.11)	8.43 (5.93)	0.90 [0.51–1.29]^∗∗∗^	0.81 [0.42–1.19]^∗∗∗^
Anxiety (STAI)^a^	45.54 (9.61)	39.26 (8.33)	37.49 (10.21)	0.65 [0.28–1.01]^∗∗∗^	0.62 [0.26–0.98]^∗∗^
Well-being (WHO-5)^a^	15.06 (3.13)	17.49 (3.06)	17.26 (3.31)	1.05 [0.63–1.46]^∗∗∗^	0.67 [0.30–1.03]^∗∗∗^

^a^*Sum scores; 95% confidence intervals of effect sizes in square brackets; CHS, Coping Humor Scale; STCI, State-Trait-Cheerfulness Inventory – state version; PSS, Perceived Stress Scale; CES-D, Center for Epidemiological Studies Depression Scale; STAI, State-Trait-Anxiety Inventory – state version; WHO-5, WHO-5 Well-Being Index; ^∗^*p* < 0.05, ^∗∗^*p* < 0.01, ^∗∗∗^*p* ≤ 0.001*.

### Aim 1: Improving Humor-Related Outcomes

Coping humor [*F*_(2,68)_ = 14.21, *p* ≤ 0.001, *d* = 1.29], cheerfulness [*F*_(2,68)_ = 19.05, *p* ≤ 0.001, *d* = 1.50], seriousness [*F*_(2,68)_ = 19.01, *p* ≤ 0.001, *d* = 1.50], and bad mood [*F*_(1.69,57.52)_ = 11.35, *p* ≤ 0.001, *d* = 1.15] showed significant main effects of time with large effect sizes. *Post hoc* tests with Bonferroni correction were also significant and effects for pre–post ranged from medium to large (*d* = 0.61 [0.24–0.96] to 0.88 [0.48–1.27]). Effects remained stable for pre–follow-up with medium to large effect sizes (*d* = 0.63 [0.26–0.99] to 0.91 [0.51–1.30]) too.

### Aim 2: Improving Mental Health-Related Outcomes

Similar results were found for mental health related outcomes: Perceived stress [*F*_(1.35,46.01)_ = 36.05, *p* ≤ 0.001, *d* = 2.06], depressiveness [*F*_(2,68)_ = 16.00, *p* ≤ 0.001, *d* = 1.37], anxiety [*F*_(1.67,56.60)_ = 10.81, *p* ≤ 0.001, *d* = 1.13], and well-being [*F*_(1.70,57.62)_ = 13.57, *p* ≤ .001, *d* = 1.26] changed significantly over time with large effect sizes. Furthermore, *post hoc* tests with Bonferroni correction were significant on all outcomes (see **Table 3**) and effect sizes ranged from medium to large with stability until follow-up (*d* = 0.62 [0.26–0.98] to 1.09 [0.66–1.50]).

### Aim 3: Evaluation of Applicability

Twenty-six participants (74.3%) completed the feedback questionnaire. Satisfaction with training (on a 1 to 5 Likert scale) was very high (*M* = 4.46, *SD* = 0.65). Understandability of the contents was rated highest (*M* = 4.85, *SD* = 0.37), whereas the improvement of symptoms lowest (*M* = 3.58, *SD* = 0.81); however, still ranging in the positive spectrum (“does partly apply” to “does rather apply”). Generally, participants would highly recommend the training (on a 1 to 4 Likert scale: *M* = 3.54, *SD* = 0.58). All means and standard deviations of the feedback items are summarized in **Table 4**.

**Table 4:** *M*, *SD* for the quantitative items of the feedback questionnaire (*n* = 26).

	*M*	*SD*
I was satisfied with the training as a whole.	4.46	0.65
The contents of the training have been well understandable for me.	4.85	0.37
The discussed topics have been interesting for me.	4.42	0.70
The structure of the sessions had a logic and comprehensible order for me.	4.46	0.51
The discussions about the humor topics were interesting.	4.23	0.86
The sharing of humor information was useful for me.	4.54	0.65
I liked the games within the humor training.	4.23	0.77
The mixture of theory and practice was convenient.	4.23	0.77
The location was comfortable.	3.73	1.08
I think I can transfer the learned in everyday life.	4.00	0.75
I can integrate humor after the training more willful in my everyday life.	3.88	0.71
Generally, I experience more cheerfulness after the training.	3.88	0.91
I think my problems have become better because of the humor training.	3.58	0.81
Would you recommend the training?^a^	3.54	0.58

*1 = does not apply at all; 2 = does rather not apply; 3 = does partly apply; 4 = does rather apply; 5 = applies completely; ^*a*^1 = no, in no case; 2 = rather not; 3 = rather yes; 4 = yes, in any case*.

Qualitative feedback revealed that participants mainly liked the group constellation and informal atmosphere (*n* = 11, 42.3%), the group leaders (*n* = 11, 42.3%), and the practical orientation of the training (*n* = 4, 15.4%). Negative feedback was primarily regarding lack of time (*n* = 3, 11.5%) and dropout/low motivation of other participants (*n* = 3, 11.5%). Furthermore, participants wanted more practical games and exercises (*n* = 6, 23.1%) and more time for the humor training in general (*n* = 5, 19.2%).

## Discussion

The present study pursued three main targets: to test the effects of a humor training on (1) humor-related outcomes, (2) mental health-related outcomes, and (3) to evaluate the applicability of the training based on the feedback of participants.

Humor-related outcomes (coping humor, cheerfulness, seriousness, and bad mood) evolved in the desired direction with medium to large effect sizes. A similar pattern of results was found for all of the mental health-related outcomes. Perceived stress, depressiveness, anxiety, and well-being showed medium to large effect sizes, too. Regarding applicability, the positive feedback of participants indicated that the training is applicable and accepted as people would recommend the training and were satisfied with it.

In the following, we want to go more into detail regarding interesting outcomes and results. First, perceived stress showed one of the strongest effects compared to all other outcomes. Time main effect, as well as *post hoc* tests, were highly significant with large effect sizes. Compared to a meta-analysis of MBSR for healthy adults (Khoury et al., 2015), the effect sizes of this study (pre–post: *d* = 1.05, pre–follow-up: *d* = 1.09) are comparable to the effect-sizes reported in the meta-analysis (pre–post: *d* = 0.83 [0.58–1.08]). One possible explanation for the strong decrease of perceived stress in this study could be explained by the improved coping humor abilities. Humor has already proven its efficacy as an adaptive way of coping with stress (Martin and Lefcourt, 1983). So, as people practiced this strategy in the training profoundly (and therefore increased in coping humor), it is not surprising that perceived stress simultaneously decreased throughout the course of training. This assumption is in line with the stability until follow-up. Participants might have continued to use coping humor until follow-up and implemented it as a preventive strategy against everyday life stress. Therefore, perceived stress continued to remain low until follow-up. However, further research with more follow-up measurements is needed, to evaluate if the relationship between coping humor and perceived stress can also be seen in the longer term. Another reason for the strong decrease of perceived stress could be due to enhanced laughter. Participants in the training had above average situations of mirth and laughter due to various games and role plays which were also transferred in daily life. Laughter is recognized as a stress-relieving process as it decreases cortisol (Berk et al., 1989), heart rate (Kraft and Pressman, 2012), and muscle tone (Paskind, 1932; Bennett and Lengacher, 2008). It might be possible that participants implemented more laughter in their everyday life and therefore had this strong decrease of stress.

Second, symptomatology like depressiveness and anxiety decreased. The positive effects of different humor interventions on depression have been already reported numerously (Beh-Pajooh et al., 2010; Crawford and Caltabiano, 2011; Gander et al., 2013; Cai et al., 2014; Proyer et al., 2014; Wellenzohn et al., 2016a,b), so the results of this study suit well with existing research and strengthen the assumption that humor can be an effective mechanism against depressive symptoms. Anxiety, however, has not been exhaustively investigated in the context of humor interventions/trainings yet. Research from non-interventional studies have already demonstrated anxiety-relieving effects of short-term induced humor (Yovetich et al., 1990; Szabo, 2003; Berk and Nanda, 2006; Ford et al., 2012). In line with these results, this study additionally shows that humor influences anxiety in the longer term, too. This assumption should be focused more in future research, testing the hypothesis that people suffering from increased anxiety or even anxiety disorders will profit from a humor intervention. There is only one study that confirms this hypothesis. Ventis et al. (2001) proved that a humorous desensitization was equally effective as a traditional systematic desensitization to reduce arachnophobia. More studies are definitely needed to evaluate the effects of humor on anxiety and anxiety disorders.

Summarizing, humor and mental health-related outcomes improved sustainably through humor training. However, one important aspect should be taken into account in future studies:

Moderating variables of humor trainings should be focused more, to be able to create a better person-intervention fit for the participants (Ruch and Hofmann, 2017). As already shown in other studies, inter-individual differences in trait cheerfulness play an important role in the effects of humor interventions (Papousek and Schulter, 2008; Hofmann et al., 2015), so it would be helpful to differentiate between high and low scorers and optimize the interventions based on these outcomes. Another personality variable which may influence the effects of humor trainings may be extraversion as extroverts experience more humor, especially benevolent humor, compared to introverts and may therefore differ in their humor behavior (Deaner and McConatha, 1993; Martin et al., 2003; Vernon et al., 2008).

Beyond inter-individual differences, another important moderator variable which should not be overlooked is group processes within the training. Humor is a social phenomenon (Martin, 2011) and inharmonic group constellations may influence the intervention’s outcome as people may not engage in the humor training as intensively as in a harmonic group constellation. Group cohesion has a powerful impact on treatment effects of group interventions (Marziali et al., 1997; Yalom and Leszcz, 2005); therefore, this moderator should be further explored. Studies including group process outcomes are still scarce; however, they are highly interesting and required to create a holistic picture about the efficacy of humor trainings.

Besides humor and mental health-related outcomes, another important part of the study was the evaluation of applicability based on feedback. Generally, participants were very satisfied with the training as 14 persons (53.8%) marked the highest score of 5 on the satisfaction item and the mean ranged between 4 and 5 (*M* = 4.46, *SD* = 0.65). Furthermore, nearly everyone, except one person, would recommend the training (*n* = 25, 92.3%). Content-related topics were evaluated consistently as positive. Especially the understandability of the training’s contents was rated highest (*M* = 4.45; *SD* = 1.24). Items about subjectively perceived change (in cheerfulness, humor, and symptoms) showed lower scores with mean values ranging from 3.5 to 4; however, they still range on the positive side of the scale.

Regarding dropout, the attrition rate of the study (17.1%) does not exceed the average attrition rate of 18.6% for group therapies of depression (McDermut et al., 2001). Also, compared to dropout in psychotherapy with up to 47% (Wierzbicki and Pekarik, 1993), the dropout rate in this study can be ranked as rather low.

As can be seen, the training was evaluated consistently positive by the majority of participants. Also, the low attrition rate pleads for the acceptance of the training. In combination with the positive outcome results, there is definitely potential to further investigate humor trainings for subclinical samples and in the longer term, maybe even implement them also in health care systems as stress-preventive programs.

### Limitations of the Study

Although results are promising, the limitations of the study should not be overlooked: First, study’s design did not include a control condition and sample size was relatively small. Due to that, findings should be interpreted with caution and should not be generalized. Further studies are needed, which include control groups and also more sophisticated designs (as randomized-controlled trials), to be able to make evaluations on the efficacy of the training. Second, follow-up was relatively short (1 month) due to restricted time and resources. But, as the results show, many outcomes continued to remain stable or even improved in the follow-up. Thus, it would be interesting to investigate the effects of the training also in further follow-ups. Third, it has to be noted that some outcomes, as anxiety and depressiveness (Clark and Watson, 1991), highly interrelate and can interact. This interaction can bias the training’s effect on outcomes. Fourth, all outcomes were assessed with state-sensitive questionnaires (either asking for the current situation or the past 2 weeks). However, it would be recommendable to assess also trait variables (e.g., trait cheerfulness) to see if the training can be effective in improving habitual outcomes sustainably. Lastly, people were thoroughly scanned in the face-to-face diagnostic interview based on SKID I and II; however, there are no objective cut-off criteria for subclinical symptoms of stress, depressiveness, anxiety, etc. in the interview. If a person reported subclinical symptoms and did not fulfill the conditions of a mental disorder concurrently, the person was invited to take part in the study. Future diagnostics should specify more concrete criteria for subclinical symptoms. However, as the interviews lasted on average at least 1 hour and the interviewers had experience in diagnostics, we assume that the lack of objective criteria for subclinical symptoms carried no big negative weight in the diagnostics because the interviewers nevertheless received a good overview of the participants’ problems.

## Conclusion

The humor training was effective in decreasing perceived stress, depressiveness, and anxiety whilst increasing coping humor, cheerfulness, and well-being in a subclinical sample. The feedback of participants was positive, indicating acceptance of the training.

However, as this was one of the first studies in this field, further research definitely is needed, including also more sophisticated designs like randomized controlled trials. Nevertheless, the results highlight the potential of humor trainings as preventive programs against stress and mental symptoms, and indicate new scopes of application, e.g., mental health care.

## Author Contributions

NT conceived and designed the work, analyzed and interpreted the data, and drafted the article. NT, VL, and ED carried out the data collection. A-RL critically revised the article.

## Conflict of Interest Statement

The authors declare that the research was conducted in the absence of any commercial or financial relationships that could be construed as a potential conflict of interest.
